# Highlight report: Role of the circadian clock system in breast cancer

**DOI:** 10.17179/excli2015-269

**Published:** 2015-04-14

**Authors:** Ahmed Ghallab

**Affiliations:** 1 Forensic Medicine and Toxicology Department, Faculty of Veterinary Medicine, South Valley University, Qena, Egypt

## ‬‬‬

It is well known that the circadian clock system coordinates physiological functions throughout the day (Dibner et al., 2010[5]; Mohawk et al., 2012[12]; Fu and Lee, 2003[6]; Hammad et al., 2013[9]). Circadian rhythms are generated by molecular feedback loops; transcriptional activators induce the expression of genes that repress their own transcription (Lowrey and Takahashi, 2011[11]). Disruption of circadian rhythms has been discussed as a possible risk factor of cancer. For example, epidemiological studies have suggested that working at night increases the risk of breast cancer (Kamdar et al., 2013[10]). However, recently a comprehensive study including 766 breast cancer patients has been published that broadens our understanding of the role of the circadian clock system in breast cancer (Cadenas et al., 2014[3]). The authors studied all known clock genes in relation to prognosis. Interestingly, loss of expression of circadian clock genes in tumour tissue was associated with a clearly higher risk to develop metastasis (Cadenas et al., 2014[3]). Recently, numerous studies have been performed to identify prognostic genes in breast cancer (e.g. Sicking et al., 2014[18][17]; Siggelkow et al., 2012[19]; Godoy et al., 2014[8]; Schmidt et al., 2008[13][14]; Cadenas, 2012;[1] Cadenas et al., 2010[2]; Ghallab, 2014[7]). However, loss of circadian clock genes seems to be of independent prognostic influence. The association of decreased circadian clock gene expression was also observed in different molecular subtypes of breast cancer (Desmedt et al., 2007[4]; Schmidt et al., 2008[13][14], 2009[16]) and was independent of established clinical factors. But the perhaps most interesting result of Cadenas et al. (2014[3]) was obtained by pairwise analysis of functionally related clock genes. PER3 and CRY2 are proteins that form dimers acting as negative feedback regulators and are known to show similar oscillation patterns. Therefore, a strong correlation between both genes can be anticipated. Indeed the authors observed strong correlations between CRY2 and PER3 in well-differentiated tumours that did not grow aggressively (Cadenas et al., 2014[3]). In contrast, the correlation between both clock genes was lost in more aggressive carcinomas. This breakdown of correlation between clock genes was also observed with loss of expression of the estrogen receptor, amplification of the oncogene HER2 and increasing histological grade (Cadenas et al., 2014[3]). In conclusion, Cadenas and colleagues have clearly shown that loss of clock gene expression and particularly the breakdown of coordinated co-expression of clock genes is associated with worse prognosis and dedifferentiation, a feature so far unknown in breast cancer.
